# Study on the Viscoelasticity Measurement of Materials Based on Surface Reflected Waves

**DOI:** 10.3390/ma12111875

**Published:** 2019-06-10

**Authors:** Jun-jie Chang, Yuan-yuan Li, Xue-feng Zeng, Hai-ying Zhong, Tao-lei Wan, Chao Lu

**Affiliations:** 1 Key Lab of Nondestructive Testing, Ministry of Education, Nanchang Hangkong University, Nanchang 330063, China; junjiechang@hotmail.com (J.-j.C.); abc997178163@gmail.com (X.-f.Z.); 13576740715@139.com (H.-y.Z.); wtl170802@163.com (T.-l.W.); luchaoniat@163.com (C.L.); 2 Yangtze Delta Region Institute of Tsinghua University, Zhejiang 314000, China; 3 Japan Probe, 1-1-14 Nakamura Chou Minami Ward Yokohama City, Kanagawa Prefecture 2320033, Japan

**Keywords:** dynamic viscoelasticity, SRM, parameter optimization, complex reflectivity

## Abstract

The reflected waves received from ultrasonic waves propagating in materials contain information that constitutes the physical properties, material composition, defects, and degradation states. When measuring the dynamic viscoelasticity, the traditional bottom reflection method (BRM) cannot be used to measure the bottom irregular samples. In this paper, the storage modulus, loss modulus, and loss tangent are extracted by the surface reflection method (SRM) to evaluate the elastomer sample viscoelasticity. A theoretical study on the phase change caused by multiple reflections in the case of non-thin layer coupling is conducted. Based on this research, the experimental system is built. The results show that considering the thickness of the coupling layer can optimize the determination of viscoelasticity and reduce the error of the viscoelastic evaluation results of an elastomer with the traditional BRM. Finally, based on the principle of the SRM, the density of the elastomers is measured, and the feasibility and overall efficiency of this method are verified by experiments.

## 1. Introduction

At present, rubber materials are widely used in transportation, the military, and national defense because of their characteristics of superelasticity, large deformation, softness, wear resistance, and insulation [1,2,3,4,5]. Rubber materials are usually used in poor environments; for example, they may be exposed to high temperatures, high pressure and high impacts when used as automobile tires, mine conveyor belts, and tank shock absorbing materials, so they are prone to fatigue and damage. As a result, rubber materials and products have strict quality requirements, such as tensile strength, modulus of elasticity, elongation, and aging resistance [6]. The internal damping mechanism is a significant evaluation index for material safety performance and to characterize it, viscoelasticity as a main mechanical property of rubber materials cannot be ignored. In order to ensure the safety of rubber materials in the course of use, the evaluation of viscoelasticity is extremely significant [7,8]. Evaluation methods of viscoelasticity mainly include dynamic mechanical analysis (DMA) [9,10], and the ultrasonic method [11]. DMA measurement requires the application of a sinusoidal alternating load. Alternatively, the conventional ultrasonic method needs to evaluate viscoelasticity through internal attenuation and sound velocity measurement of the material is essential [12,13], which requires cutting the test sample. Consequently, these methods are very inconvenient to carry out in the field, and the requirements on the bottom state of the material are too high in terms of the shape of the free bottom surface. In this paper, from industrial applicability, the surface reflection method (SRM) is used instead of the traditional bottom reflection method (BRM) [14,15]. Based on the theory of elasticity and viscosity, the theory of the viscoelasticity evaluation method is researched. According to the research of material properties such as the storage modulus $L^{\prime }$ and loss modulus L″, the value of tanδ, which has a great relationship with friction, was used in the viscoelastic index, and following simulation and experiment, the SRM was shown to be feasible. Typically, the SRM and BRM ignore the thickness of the coupling layer; however, it was found that adding the influence of coupling layer thickness to the calculation can greatly reduce the error [16,17]. The density measurement must be performed by the Archimedes’ principle in these measurements, which is complicated. Therefore, a simple surface density measurement method using ultrasonic waves is discussed. Additionally, the accuracy was confirmed by experiments and the practical obstacle of the ultrasonic evaluation method of viscoelasticity was eliminated.

## 2. SRM Theory

### 2.1. Viscoelasticity Evaluation

The elasticity of an ideal elastic body obeys Hooke’s law [18,19], where stress ε is proportional to strain σ. Here, E is Young’s modulus:

(1) σ=Eε

The viscosity of ideal liquids is subordinated to Newton’s law [20,21]; that is, the stress ε is proportional to the strain rate, and the proportional coefficient η is the viscosity:

(2) $$\sigma =\eta \frac{d\varepsilon }{dt}$$

After pure viscous fluid is subjected to an external force, flow is generated, and all the energy is converted into kinetic energy. This is not stored [14,22], even in the case of eliminating external forces, and the original state before the force was applied cannot be restored; pure elastic solids store all the energy after being subjected to an external force. After removing the external force, the material returns to the original state before the force is applied [23]. The viscoelastic material simultaneously consumes and stores energy, and thus has flow characteristics and elasticity. When subjected to external forces, it can store part of the energy and produce partial flow. This part of the energy is released after removing the external force, and the material is recovered, but cannot be completely restored to the state it was in before the force was applied [24,25].

There are two kinds of research on the viscoelastic properties of materials: one is static viscoelasticity research and the other is dynamic viscoelasticity research [26]. Among them, static viscoelasticity is a viscoelastic property of materials in the process of analyzing the static mechanical behavior and dynamic viscoelasticity is the viscoelasticity of materials in the process of dynamic mechanical behavior. Therefore, dynamic viscoelasticity is a better reflection of the performance of materials under actual conditions of use. In the process of dynamic mechanical behavior analysis, two phenomena can be observed: hysteresis and mechanical loss. The main parameters of dynamic viscoelasticity are the storage modulus, loss modulus, and loss tangent [27,28].

**Figure 1:**
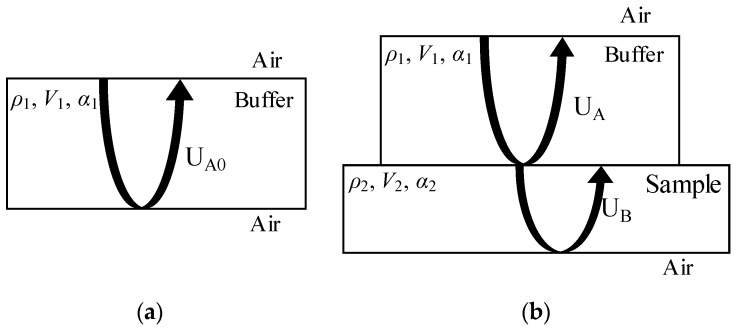
Schematic diagram of the surface reflection measurement principle for measuring the complex reflection coefficient: (**a**) with no sample: reflected wave A_0_ of buffer and the air interface; (**b**) with a sample: reflected wave A of buffer and the sample interface, and reflected wave B of sample and the air interface.

In dynamic mechanical experiments, sinusoidal stress is the most common alternating stress. A stress expression using dynamic shear as an example is as follows:

(3) $$\sigma_{\tau }\left(t\right)=\sigma_{\tau 0}\sin\omega t$$

where $\sigma_{\tau 0}$ is the stress amplitude, $\gamma_{0}$ is the strain amplitude, and ω is the angular frequency. Due to the nature of the material, the strain response of the material under sinusoidal alternating stress is shown in Figure 1. For an ideal elastomer, the response of the strain to the stress is instantaneous, so the strain response is a sinusoidal function in-phase with the stress as follows:

(4) $$\gamma \left(t\right)=\gamma_{0}\sin\omega t$$

The relationship between the stress and strain of an ideal viscous material is as shown in Expression (2).

(5) $$\gamma \left(t\right)=\gamma_{0}\sin\left(\omega t-90^{{}^{\circ}}\right)$$

The strain lags behind the stress by 90°. For viscoelastic materials, the strain lags behind the stress by a phase angle δ (0° < δ < 90°); namely, the stress occurs earlier than the strain by a phase angle. Strain and stress are given by:

(6) $$\varepsilon_{\tau }\left(t\right)=\varepsilon_{0}\sin\omega t$$

(7) $$\sigma_{\tau }\left(t\right)=\sigma_{\tau 0}\sin\left(\omega t+\delta \right)$$

When expanded into two parts,

(8) $$\sigma \left(t\right)=\sigma_{0}\sin\omega t\;\cos\;\delta +\sigma_{0}\cos\omega t\;\sin\;\delta$$

where $\sigma_{0}\sin\omega t\;\cos\;\delta$ is the stress in phase with strain and belongs to the main power of elastic deformation and $\sigma_{0}\cos\omega t\sin\delta$ is the stress that is 90° out of strain, which corresponds to the viscous deformation, and the frictional resistance can be overcome when consumed.

If L′ is defined as the ratio of the amplitude of the stress and the strain in-phase, L″ is the ratio of the stress amplitude to the strain amplitude which is 90° out of phase.

(9) $$L^{\prime }=\frac{\sigma_{0}\cos\delta }{\varepsilon_{0}}=\frac{\sigma_{\infty }}{\varepsilon_{0}}\cos\delta$$

(10) $$L^{\prime \prime }=\frac{\sigma_{0}\sin\delta }{\varepsilon_{0}}=\frac{\sigma_{0}}{\varepsilon_{0}}\sin\delta$$

Available from Equations (7)–(9):

(11) $$\sigma \left(t\right)={L^{\prime }\varepsilon }_{0}\sin\omega t+{L^{\prime \prime }\varepsilon }_{0}\cos\omega t$$

Therefore, it can be obtained from the mathematical plural form that:

(12) $$L^{*}=L^{\prime }+iL^{\prime \prime }$$

where $L^{*}$ is the complex modulus; $L^{\prime }$ prime is the real modulus, that is, the storage modulus, which is used to store the heat of the elastic deformation of the material’s deformation process; and $L^{\prime \prime }$ is the imaginary modulus, namely the loss modulus, which is used to reflect the heat lost during the deformation of the material. According to the above description, the properties of the viscoelastic material are between a pure elastic solid and a pure viscous fluid, and the strain and stress generated in it are not synchronized. The phase angle difference between the two is δ (0° < δ < 90 °), which is called the loss angle. Its tangent value is calculated as:

(13) $$\tan\delta =\frac{L^{\prime \prime }}{L^{\prime }}$$

Therefore, the loss angle δ can be used to evaluate the viscoelastic properties of the material.

Baker [29] used the shear wave to evaluate the viscoelasticity of the liquid by the SRM at an early stage. In addition, Morozov, Joshi and Kumar et al. [30,31] used the longitudinal wave to study the complex acoustic impedance measurement. The accuracy is improved by standardizing the measured values of the samples based on the reflectance of the air and the buffer material. In this paper, the SRM is applied to rubber as a viscoelastic solid instead of a viscous fluid, and an attempt is made to calculate the loss tangent value tanδ.

The measurement model of the complex reflectance of the surface reflection is shown as Figure 1. For the buffer, the acoustic impedance is set to *Z*_1_, the sound speed is set to *V*_1_, the density is set to $\rho_{1}$, the attenuation coefficientis set to α_1_, the reflectance from the buffer to the air is set to *R*_1_, and the reflected wave A_0_ amplitude is set to U_A0_. For the sample, the acoustic impedance is set to *Z*_2_, the sound velocity is set to *V*_2_, the density is set to $\rho_{2}$, the attenuation coefficientis set to α_2_, the reflectance from the buffer to the sample is assumed to be *R*_2_, the amplitude of the reflected wave A is set to U_A_, and the amplitude of the reflected wave B is set to U_B_. The measuring method and device are the same as those employed in the BRM.

The calculation of viscoelasticity of the surface reflected wave is made by considering the complex reflectance. When the influence of the coupling dielectric layer between the buffer and the sample is ignored, the acoustic impedance is used to represent the complex reflectivity *R*_2_ from the buffer to the sample.

(14) $$R_{2}=\frac{z_{2}-z_{1}}{z_{2}+z_{1}}$$

Here, *Z*_1_ is the buffer acoustic impedance and *Z*_2_ is the acoustic impedance of the sample. From Equation (14):

(15) $$Z_{2}=\frac{1+R_{2}}{1-R_{2}}Z_{1}$$

where

(16) $$R_{2}=-\frac{\left|A\right|e^{i\theta_{A}}}{\left|A_{0}\right|e^{i\theta_{A}}}=-\left|R_{2}\right|e^{-i\theta }\;\left|R_{2}\right|=\frac{\left|A\right|}{\left|A_{0}\right|}\;\theta =\theta_{A_{0}}-\theta_{A}$$

Due to the phase angle $\theta_{A_{0}},\theta_{A}$ will produce an arbitrariness of 2Nπ, and 2Nπ will be added at any time to form a linear correction.

(17) $$Z_{2}=\frac{1-\left|R_{2}\right|e^{-i\theta }}{1+\left|R_{2}\right|e^{-i\theta }}Z_{1}$$

*Z*_2_ can be set to the following complex form:

(18) $$Z_{2}=X+Yi$$

Then,

$$X=\frac{1-{\left|R_{2}\right|}^{2}}{1+2\left|R_{2}\right|\cos\theta +{\left|R_{2}\right|}^{2}}Z_{1}$$

$$Y=\frac{2\left|R_{2}\right|\sin\theta }{1+2\left|R_{2}\right|\cos\theta +{\left|R_{2}\right|}^{2}}Z_{1}$$

Therefore, the complex modulus has the following relationship

(19) $$L^{*}=L^{\prime }+iL^{\prime \prime }=\frac{Z_{2}{}^{2}}{\rho_{2}}$$

Because

(20) $$\frac{Z_{2}{}^{2}}{\rho_{2}}=\frac{X^{2}-Y^{2}+2XYi}{\rho_{2}}$$

Therefore, the storage modulus and the loss modulus are as follows:

(21) $$L^{\prime }=\frac{X^{2}-Y^{2}}{\rho_{2}}=\frac{Z_{1}{}^{2}}{\rho_{2}}\frac{{\left(1-{\left|R_{2}\right|}^{2}\right)}^{2}-4{\left|R_{2}\right|}^{2}\sin^{2}\theta }{{\left(1+2\left|R_{2}\right|\cos\theta +{\left|R_{2}\right|}^{2}\right)}^{2}}$$

(22) $$L^{\prime \prime }=\frac{2XY}{\rho_{2}}=\frac{Z_{1}{}^{2}}{\rho_{2}}\frac{4\left|R_{2}\right|\left(1-{\left|R_{2}\right|}^{2}\right)\sin\theta }{{\left(1+2\left|R_{2}\right|\cos\theta +{\left|R_{2}\right|}^{2}\right)}^{2}}$$

The loss tangent value can be obtained from Equation (23):

(23) $$\tan\delta =\frac{L^{\prime \prime }}{L^{\prime }}=\frac{4\left|R_{2}\right|\left(1-{\left|R_{2}\right|}^{2}\right)\sin\theta }{{\left(1-{\left|R_{2}\right|}^{2}\right)}^{2}-4{\left|R_{2}\right|}^{2}\sin^{2}\theta }$$

where:

(24) $$Z_{1}=\rho_{1}V_{1}$$

Therefore, without considering the thickness of the coupling layer and the attenuation of the buffer, if the acoustic impedance *Z*_1_ of the buffer (or density $\rho_{1}$, sound velocity *V*_1_) is obtained, the time domain signal and frequency domain of A_0_ and A are experimentally measured and the material viscoelasticity can be evaluated.

### 2.2. Influence of the Coupling Layer

**Figure 2:**
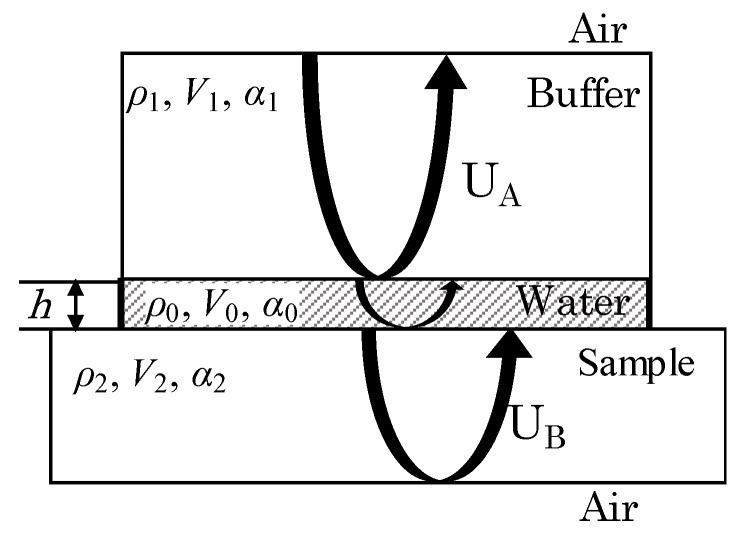
Effect of the coupling layer: coupling layer thickness is *h* and the medium is water.

In the process of acoustic coupling between the buffer and the sample, a coupling layer with a certain thickness will appear, and the model with the coupling layer is shown in Figure 2. It is assumed that the model’s coupling agent is water. The thickness is represented by *h*, and the density, sound velocity, and attenuation coefficient of the water coupling layer are represented by ρ_0_, *V*_0_, and α_0_ respectively. α_1_ is the attenuation coefficient of the buffer and α_2_ is the attenuation coefficient of the sample.

When the thickness *h* of the coupling layer is sufficiently large relative to the wavelength λ, multiple reflections in the coupling layer cause phase changes, which is worth exploring. When considering the thickness of the coupling medium (water), the complex reflectivity calculation equation based on the surface method is used, assuming that the acoustic coupling between the buffer, the water and the sample is ideal (displacement, continuous stress). At this time, the complex reflectance of the harmonic wave of the angular frequency ω is as follows:

(25) $$R_{2}=\frac{\left(1+\alpha^{2}\right)z_{0}\left(z_{1}-z_{2}\right)-\left(1-\alpha^{2}\right)\left(z_{0}^{2}-z_{1}z_{2}\right)}{\left(1+\alpha^{2}\right)z_{0}\left(z_{1}+z_{2}\right)+\left(1-\alpha^{2}\right)\left(z_{0}^{2}+z_{1}z_{2}\right)}\;\alpha =\exp(ik_{0}h)$$

where:

(26) $$k_{0}=\frac{\omega }{V_{0}}+i\alpha_{0}=\frac{\omega }{V_{0}}$$

(27) $$k_{1}=\frac{\omega }{V_{1}}+i\alpha_{1}=\frac{\omega }{V_{1}}$$

Ignoring the attenuation of water and buffer, the calculation is made according to $\alpha_{0}=0,{\;\alpha }_{1}=0$:

$$z_{0}=\rho_{0}V_{0}$$

$$z_{1}=\rho_{1}V_{1}$$

Deformation of *Z*_2_ is calculated as follows:

(28) $$z_{2}=\frac{2z_{0}z_{1}\left(1-R^{2}\right)+i\left[\left(z_{0}^{2}-z_{1}^{2}\right)\sin\varphi +\left\{{\left(z_{0}+z_{1}\right)}^{2}\sin\left(\theta +\varphi \right)-{\left(z_{0}-z_{1}\right)}^{2}\sin\psi \right\}R+\left(z_{0}^{2}-z_{1}^{2}\right)R^{2}\sin\varphi \right]}{\left\{z_{0}^{2}+z_{1}^{2}+\left(z_{0}^{2}-z_{1}^{2}\right)\cos\varphi \right\}+\left\{{\left(z_{0}+z_{1}\right)}^{2}\cos\left(\theta +\varphi \right)+2\left(z_{0}^{2}-z_{1}^{2}\right)\cos\theta +{\left(z_{0}-z_{1}\right)}^{2}\cos\psi \right\}R+\left\{z_{0}^{2}+z_{1}^{2}+\left(z_{0}^{2}-z_{1}^{2}\right)\cos\varphi \right\}R^{2}}z_{0}$$

Then, the real part is set to *X* and the imaginary part is set to *Y*:

(29) $$z_{2}=X+iY\;X=\frac{2z_{0}z_{1}\left(1-R^{2}\right)}{\left\{z_{0}^{2}+z_{1}^{2}+\left(z_{0}^{2}-z_{1}^{2}\right)\cos\varphi \right\}+\left\{{\left(z_{0}+z_{1}\right)}^{2}\cos\left(\theta +\varphi \right)+2\left(z_{0}^{2}-z_{1}^{2}\right)\cos\theta +{\left(z_{0}-z_{1}\right)}^{2}\cos\psi \right\}R+\left\{z_{0}^{2}+z_{1}^{2}+\left(z_{0}^{2}-z_{1}^{2}\right)\cos\varphi \right\}R^{2}}z_{0}\;Y=\frac{\left[(z_{0}^{2}-z_{1}^{2})\sin\varphi +\{{\left(z_{0}+z_{1}\right)}^{2}\sin\left(\theta +\varphi \right)-{\left(z_{0}-z_{1}\right)}^{2}\sin\psi \}R+(z_{0}^{2}-z_{1}^{2})R^{2}\sin\varphi \right]}{\left\{z_{0}^{2}+z_{1}^{2}+\left(z_{0}^{2}-z_{1}^{2}\right)\cos\varphi \right\}+\left\{{\left(z_{0}+z_{1}\right)}^{2}\cos\left(\theta +\varphi \right)+2\left(z_{0}^{2}-z_{1}^{2}\right)\cos\theta +{\left(z_{0}-z_{1}\right)}^{2}\cos\psi \right\}R+\left\{z_{0}^{2}+z_{1}^{2}+\left(z_{0}^{2}-z_{1}^{2}\right)\cos\varphi \right\}R^{2}}z_{0}$$

The elasticity relationship is as follows:

(30) $$L^{*}=L^{\prime }+iL^{\prime \prime }=\frac{Z_{2}{}^{2}}{\rho_{2}}$$

where:

(31) $$L^{\prime }=\frac{X^{2}-Y^{2}}{\rho_{2}}=\frac{z_{0}^{2}}{\rho_{2}}\frac{{\left[4z_{0}^{2}z_{1}^{2}{\left(1-R^{2}\right)}^{2}-\left[\left(z_{0}^{2}-z_{1}^{2}\right)\sin\varphi +R\left\{{\left(z_{0}+z_{1}\right)}^{2}\sin\left(\theta +\varphi \right)-{\left(z_{0}-z_{1}\right)}^{2}\sin\psi \right\}+\left(z_{0}^{2}-z_{1}^{2}\right)R^{2}\sin\varphi \right]\right]}^{2}}{{\left[\left\{z_{0}^{2}+z_{1}^{2}+\left(z_{0}^{2}-z_{1}^{2}\right)\cos\varphi \right\}+\left\{{\left(z_{0}+z_{1}\right)}^{2}\cos\left(\theta +\varphi \right)+2\left(z_{0}{}^{2}-z_{1}^{2}\right)\cos\theta +{\left(z_{0}-z_{1}\right)}^{2}\cos\psi \right\}R+\left\{z_{0}^{2}+z_{1}^{2}+\left(z^{2}-z^{2}\right)\cos\varphi \right\}R^{2}\right]}^{2}}$$

(32) $$L^{\prime \prime }=\frac{2XY}{\rho^{2}}=\frac{z_{0}^{2}}{\rho_{2}}\frac{4z_{0}z_{1}\left(1-R^{2}\right)\left[\left(z_{0}^{2}-z_{1}^{2}\right)\sin\varphi +\left\{{\left(z_{0}+z_{1}\right)}^{2}\sin\left(\theta +\varphi \right)-{\left(z_{0}-z_{1}\right)}^{2}\sin\Psi \right\}R+\left(z_{0}^{2}-z_{1}^{2}\right)R^{2}\sin\varphi \right]}{{\left[\left\{z_{0}^{2}+z_{1}^{2}+\left(z_{0}^{2}-z_{1}^{2}\right)\cos\varphi \right\}+\left\{{\left(z_{0}+z_{1}\right)}^{2}\cos\left(\theta +\varphi \right)+2\left(z_{0}{}^{2}-z_{1}^{2}\right)\cos\theta +{\left(z_{0}-z_{1}\right)}^{2}\cos\psi \right\}R+\left\{z_{0}^{2}+z_{1}^{2}+\left(z^{2}-z^{2}\right)\cos\varphi \right\}R^{2}\right]}^{2}}$$

The loss tangent given as:

(33) $$\tan\delta =\frac{L^{\prime \prime }}{L^{\prime }}=\frac{2z_{0}z_{1}\left(1-R^{2}\right)\left[\left(z_{0}^{2}-z_{1}^{2}\right)\sin\varphi +\left\{{\left(z_{0}+z_{1}\right)}^{2}\sin\left(\theta +\varphi \right)-{\left(z_{0}-z_{1}\right)}^{2}\sin\Psi \right\}R+\left(z_{0}^{2}-z_{1}^{2}\right)R^{2}\sin\varphi \right]}{{\left[\left\{z_{0}^{2}+z_{1}^{2}+\left(z_{0}^{2}-z_{1}^{2}\right)\cos\varphi \right\}+\left\{{\left(z_{0}+z_{1}\right)}^{2}\cos\left(\theta +\varphi \right)+2\left(z_{0}{}^{2}-z_{1}^{2}\right)\cos\theta +{\left(z_{0}-z_{1}\right)}^{2}\cos\psi \right\}R+\left\{z_{0}^{2}+z_{1}^{2}+\left(z^{2}-z^{2}\right)\cos\varphi \right\}R^{2}\right]}^{2}}$$

where: $z_{0}=\rho_{0}c_{0}$, $z_{1}=\rho_{1}c_{1}$, $R=\left|R_{2}\right|=\left|\frac{A}{A_{0}}\right|$, $\theta =\theta_{A_{0}}-\theta_{A}$, $\varphi =2k_{0}h=\frac{2\omega h}{V_{0}}=\frac{4\pi fh}{c_{0}}$, ψ=θ−φ.

Therefore, in the case of considering the thickness of the coupling layer, if the acoustic impedance *Z*_0_ of the coupling layer (or density $\rho_{0}$, sound velocity *V*_0_), the acoustic impedance *Z*_1_ of the buffer (or density $\rho_{1}$, sound velocity *V*_1_), the time domain signal, and the frequency domain signal of the received signals A_0_ and A are all known, the viscoelasticity of the sample can be evaluated. In this paper, the viscoelastic evaluation of rubber materials with different compositions is carried out by simulation and experimental verification. The results when considering the thickness of the coupling layer or without considering the thickness of the SRM are compared with the results of the conventional BRM and the error is analyzed.

## 3. Viscoelastic Evaluation and Optimization

### 3.1. Comparison with Traditional DMA Method

In order to verify the correctness of the viscoelastic evaluation result of the ultrasonic method, the viscoelasticity of the same kind of rubber polymer material was evaluated using the BRM and the DMA method, the loss tangent tan δ was used to characterize the viscoelasticity of materials [11,32,33].

The DMA method experimental device is a Rheogel-E4000 dynamic mechanical analyzer provided by UBM Corporation of Tokyo, Japan. A sinusoidal strain vibration with an amplitude of 5 μs is applied to the experimental material, and the strain waveform is applied as shown in Equation (4). The material loss tangent tan δ was obtained in a tensile mode. Unlike the DMA method, in dynamic viscoelastic evaluation, the ultrasonic pulse reflection method does not require a dynamic alternating stress, so it does not cause damage to the sample. The BRM used an ultrasonic probe with a central frequency of 1 MHz. Fast Fourier transform (FFT) is performed on the received time domain waveform and the loss tangent tan δ is calculated.

**Figure 3:**
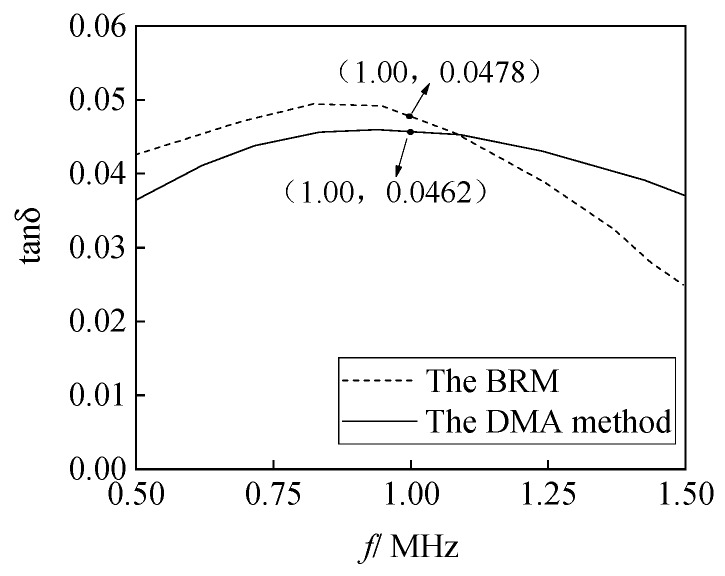
Loss tangent curve of the dynamic mechanical analysis (DMA) method and the bottom reflection method (BRM) in the effective frequency range of 0.5–1.5 MHz.

The value of tan δ is dependent on frequency, so the BRM is compared with the DMA method in the range of ultrasonic bandwidth, which makes the results more convincing [32]. As shown in Figure 3, the loss tangent tan δ results of the BRM and DMA method are consistent at the center frequency 1 MHz and the error is 3.4%, which proves the reliability of the evaluation results of the BRM.

**Figure 4:**
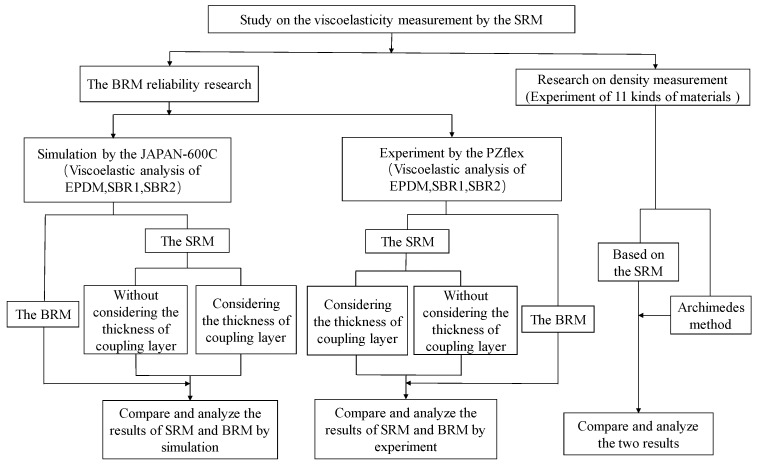
The flowchart of the process adopted for research on viscoelasticity. EPDM = ethylene propylene diene monomer; SBR = styrene butadiene rubber.

The BRM was compared with the traditional DMA method, which proved the reliability of the evaluation result of the ultrasonic BRM. The SRM is an improvement of the BRM, therefore, in this article, it will be compared with the results of the BRM in the latter study. A flowchart of the process adopted for the research is shown in Figure 4.

### 3.2. Simulation

**Figure 5:**
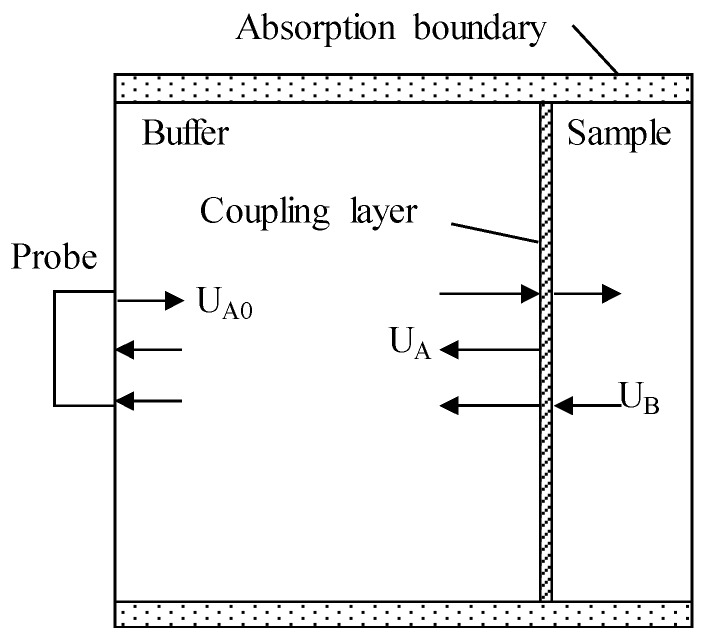
Schematic diagram of the simulation model by the PZflex method and wave propagation process with 50 μm-thick aqueous medium and absorption boundary.

**Figure 6:**
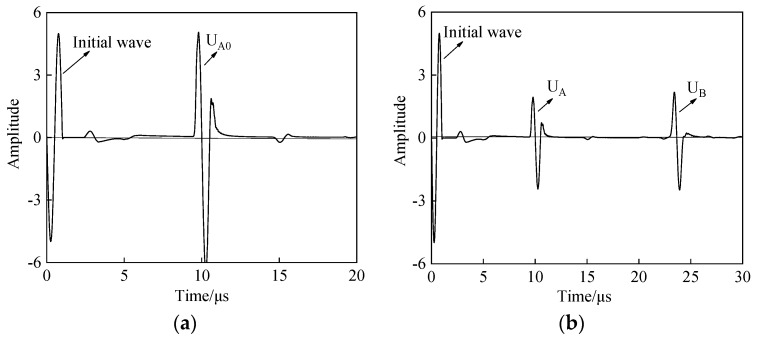
Received waveform of the same 13 mm-thick polymethyl methacrylate (PMMA) and 10 mm-thick EPDM material (1 MHz): (**a**) the reflection wave from the bottom of the buffer with simulation; (**b**) the reflection wave from the surface and bottom of the sample with simulation.

The model built using PZflex analysis software (version x) is shown in Figure 5, the upper and lower boundary conditions are absorption boundaries, and the materials used for the simulation are three kinds of practical rubbers: ethylene propylene diene monomer (EPDM) and two kinds of styrene butadiene rubber (SBR1 and SBR2). All sample thicknesses are set to 10 mm. The buffer material is polymethyl methacrylate (PMMA) with a thickness of 13 mm. The sensor size is 12 mm, and the detection frequency is set to 1 MHz. In the absence of a sample, the reflected wave A_0_ of the buffer bottom surface is obtained, and the amplitude is U_A0_. The sample is placed at the end of the buffer. Then the sample–buffer aqueous medium coupling layer is set to 50 μm, and the sample surface reflected wave A and bottom surface reflected wave B are obtained. The amplitudes are represented as U_A_ and U_B_, respectively. Taking EPDM material as an example, the receiving waveform in the simulation is shown in Figure 6.

**Figure 7:**
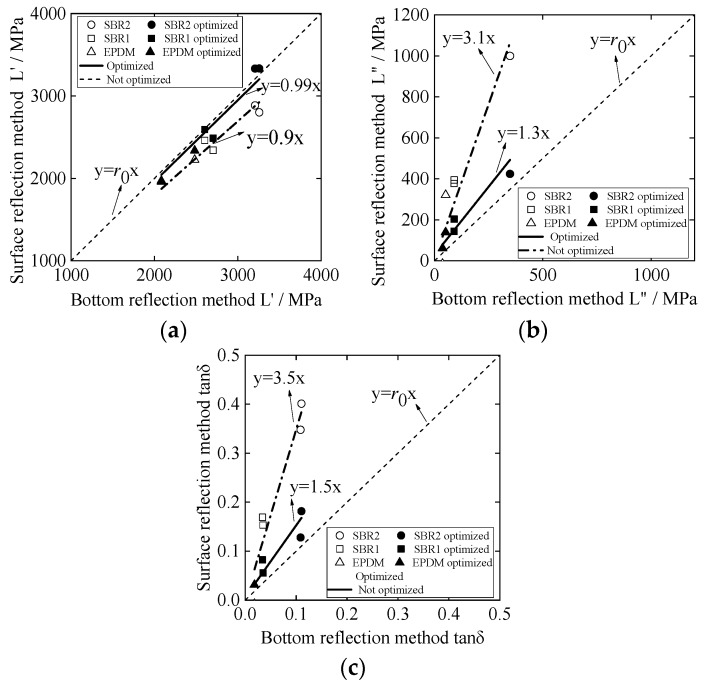
Linear approximation of the SRM and BRM simulation results for different kinds of rubber materials: (**a**) analysis of storage modulus $L^{\prime }$ results; (**b**) analysis of loss modulus $L^{\prime \prime }$ results; (**c**) analysis of loss tangent tanδ results.

Figure 7 shows the storage modulus $L^{\prime }$, the loss modulus $L^{\prime \prime }$, and the loss tangent tan δ calculated from the rubber surface reflectance. According to the principle of the traditional BRM, $L^{\prime }$, $L^{\prime \prime }$, and tan δ of the three materials are calculated [14]. According to the theoretical analysis in Section 1, $L^{\prime }$, $L^{\prime \prime }$, and tan δ of different rubber materials are solved, respectively. The effect of the thickness *h* of the water layer is added to optimize the results. The results of the BRM and SRM are compared by linear approximation, as shown in Figure 7a–c. The *x*-axis represents the evaluation result of the BRM, and the *y*-axis represents the evaluation result of the SRM. Based on $y=r_{0}x$ (where *r_0_* is correlation coefficient and *r*_0_ = 1), a linear approximation of the two results is conducted considering the thickness of the coupling layer (water). Comparing the correlation coefficient *r* with the proximity of *r*_0_, when *r* >1, the higher the *r* value, the higher the measured value of the SRM compared to the BRM. However when *r* < 1, the smaller the *r* value, the higher the measured value of the BRM compared to the SRM. The closer the *r* value is to 1, the better the fit of the two results. This comparison method can visually indicate the feasibility of the SRM and the optimization degree of the result considering the thickness of the water layer.

The hollow marks indicate the result without considering the thickness of the water layer, the solid marks indicate the optimized result, and the linear regression equation is obtained separately [34,35]. For the storage modulus $L^{\prime }$, from the comparison of the results in Figure 7a, the optimized result is more fitting with $y=r_{0}x$, and the correlation coefficient *r*_1_ = 0.9 when the thickness of the water layer is not considered; however, the correlation coefficient of the linear regression equation is *r*_2_ = 0.99 after optimization is obtained. The analysis shows that the storage modulus $L^{\prime }$ obtained by the SRM is basically the same as that of the BRM but has some error; by adding the coupling layer thickness to the optimization calculation method, the errors of the two evaluation methods are reduced. For the loss modulus $L^{\prime \prime }$, from the comparison of the results in Figure 7b, the result of the optimized front SRM is much larger than the BRM, and at this time, the error is large, with a value of *r* = 3.1. When considering the water layer thickness, the error is greatly reduced, and after optimization, *r* = 1.5, making the SRM measurement result more feasible. For the loss tangent tanδ for the viscoelasticity of materials, the comparison result is shown in Figure 7c, similar to Figure 7b, using the SRM and the BRM to evaluate the same material. When ignoring the thickness of the coupling layer between the buffer and sample, the SRM results are higher than those of the BRM, but the increase or decrease of the viscoelasticity of different materials has the same reference value and has some reference value. To reduce errors, when the thickness of the coupling layer between the buffer and sample is taken as the influencing factor, the SRM is slightly higher than the BRM, and the error of the evaluation results of the viscoelasticity of the material with the BRM is reduced. Thus, the optimization effect is better.

### 3.3. Experimental Verification

**Figure 8:**
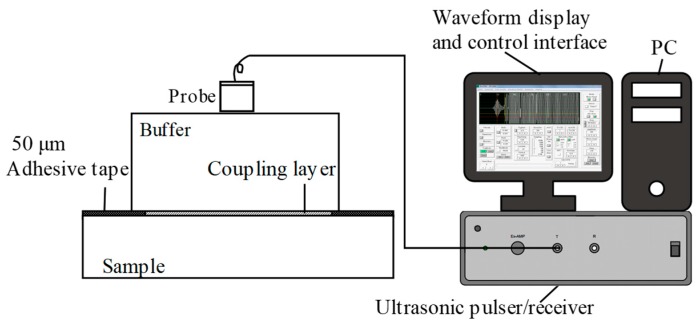
Schematic diagram of the experimental setup.

**Figure 9:**
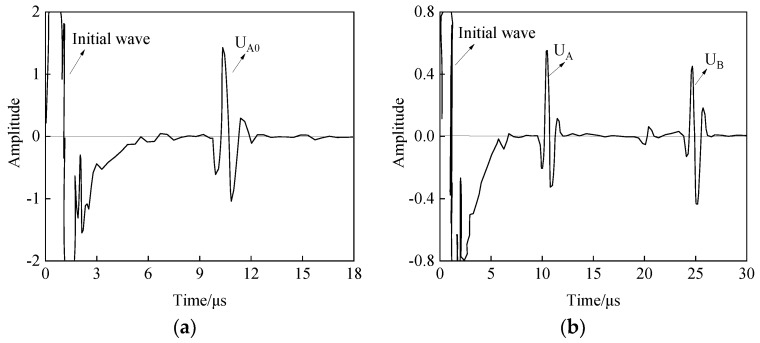
Received waveform of the same 13 mm-thick PMMA and 10 mm-thick EPDM material (1 MHz) by experiment: (**a**) the reflection wave form of the bottom of the buffer with an experiment; (**b**) the reflection wave form of the surface and bottom of the sample with an experiment.

The experimental system employed to measure verification is shown in Figure 8. A JAPAN-600C ultrasonic pulser/receiver (Japan Probe Co. Ltd, Yokohama, Japan) is used in the experiment. The transmitting–receiving transducer was provided by Japan Probe Co. Ltd (Yokohama, Japan), and has a central frequency of 1 MHz and a 12 mm circular wafer. In order to control the thickness of the coupling layer in the experiment, 50 μm-thick polyimide tape is applied between the buffer layer and sample, and the tape is only pasted at the edges on both sides of the buffer layer. The coupling agent is used between the buffer layer and the sample. Then, force extrusion is used to ensure that the thickness of the coupling layer is close to the thickness of the tape. Consistent with the simulation, three kinds of practical rubber are used as sample materials: EPDM, SBR1, and SBR2. The thickness of the samples is 10 mm. The buffer material is PMMA with a thickness of 13 mm. Taking EPDM as an example, the received waveform in the experiment is shown in Figure 9.

The received waveforms are similar to the simulation results in the experiment, but more chaotic than the simulated receiving waves since the buffer material damping coefficient is not 0 in reality and the coupling state is ideal in the simulation process but not in the experiment.

**Figure 10:**
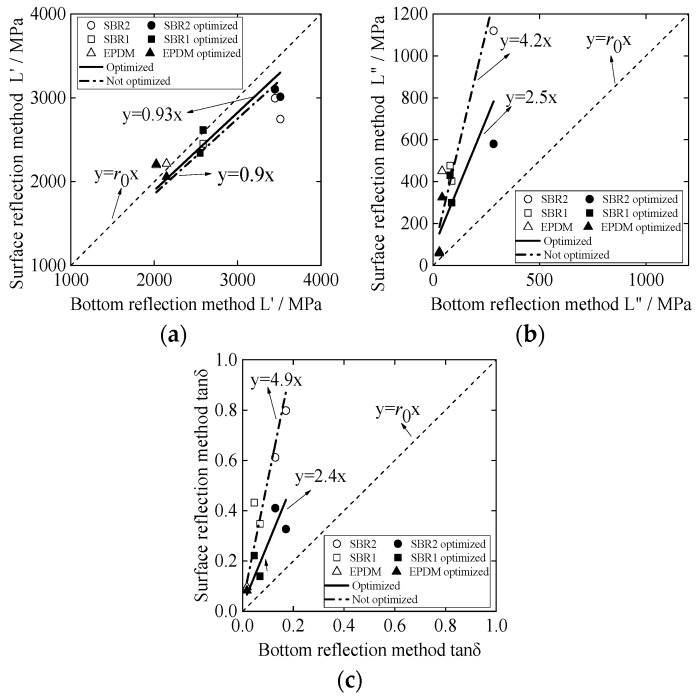
Linear approximation of the SRM and BRM experimental results for different kinds of rubber materials: (**a**) analysis of storage modulus $L^{\prime }$ results; (**b**) analysis of loss modulus $L^{\prime \prime }$ results; (**c**) analysis of loss tangent tanδ results.

Figure 10a,b shows the relationship between the SRM results and BRM results for storage modulus $L^{\prime }$ and loss modulus $L^{\prime \prime }$, respectively, which included the following situations: with optimization (considering the thickness of the coupling layer) or without optimization (not considering the thickness of the coupling layer). Through comparing correlation coefficients, after optimization, the value *r* for $L^{\prime }$ shifted from 0.9 to 0.93, while value *r* for $L^{\prime \prime }$ shifted from 4.2 to 2.5. This phenomenon is almost consistent with the simulation result shown in Figure 7, and the optimized results are more fitting with $y=r_{0}x$. The loss tangent tan δ, which can be used to evaluate the viscoelasticity of the material, is calculated according to the SRM and the BRM, respectively. Figure 10c shows the results of the evaluation of the same kind of materials by the SRM and the BRM. Before optimization, the correlation coefficient was *r* = 4.9 and the SRM‘s results are higher than the BRM‘s; after optimization, the SRM result is slightly higher than the BRM’s, and *r* = 2.4, which reduces the error of the evaluation result of the material viscoelasticity with the BRM. Compared with the simulation analysis results in Figure 7c, although it is proved that the study of the thickness of the coupling layer can optimize the viscoelastic evaluation of the material, the simulation results are better than the experimental results. In the SRM, although the phase difference is the main measurement factor, same other error factors of the conventional BRM can also be considered, such as the matching of the acoustic impedance of the buffer material and the sample and the close-range sound field caused by the length of the buffer material. Physical properties such as deterioration of the surface of the sample, change from the surface toward the deep portion, and the presence of bubbles inside the sample are also considered, which may be factors of error. This will serve as a research topic for the future.

## 4. Application of Density Measurement by the SRM

### 4.1. Theoretical Analysis

In the measurement of the material properties such as the storage modulus $L^{\prime }$ and the loss elastic modulus $L^{\prime \prime }$ by ultrasonic waves, the measurement of density is indispensable, but the traditional method is time-consuming and laborious [36,37]. The density of the corresponding unknown material ρ can be measured by the relationship between the volume v and the mass m.

(34) $$\rho =\frac{m}{v}$$

Therefore, if the density is measured by ultrasonic waves, the efficiency of material property measurement can be greatly improved. This method is studied, which uses the SRM to measure the acoustic impedance and speed of sound to determine the density, and also sets the acoustic impedance of the buffer to *Z*_1_, the sound velocity to *V*_1_, and the density to ρ_1_. From the buffer to the air, the reflectance is set to *R*_1_, the acoustic impedance of the sample is set to *Z*_2_, the sound velocity is set to *V*_2_, and the density is set to ρ_2_. Assuming that the reflectance from the buffer to the sample is *R*_2_, the acoustic impedance *Z*_2_ of the sample is obtained by the following equation:

(35) Z=ρV

(36) $$R_{2}=\frac{Z_{2}-Z_{1}}{Z_{2}+Z_{1}}$$

Equation (35) is deformed as follows:

(37) $$Z_{2}=\frac{1+R_{2}}{1-R_{2}}Z_{1}$$

The reflection with the air is the total reflection:

(38) $$R_{1}=\frac{A_{0}}{A^{*}}=-1$$

(39) $$A^{*}=-A_{0}$$

(40) $$R_{2}=\frac{A}{A^{*}}=-\frac{A}{A_{0}}$$

In addition,

(41) $$Z_{1}=\rho_{1}V_{1}$$

Since the density ρ_1_ and the sound velocity *V*_1_ of the buffer are known, then *Z*_2_ of the sample is obtained. Therefore, the density ρ_2_ of the sample can be obtained from the bottom surface reflected wave of the sample and the sound velocity *V*_2_ obtained from the surface reflection.

(42) $$\rho_{2}=Z_{2}/V_{2}$$

#### 4.2. Theoretical Analysis

In practical applications, the traditional method of measuring density required cutting the test sample, however, it is usually impossible to cut it into a regular shape which can be calculated by volume formulas. The Archimedes method is a density measurement method based on Equation (34) that converts volume measurement into mechanical measurement. In this experiment, a sample of arbitrary shape is cut from the rubber material to be tested. After being tied with a string, the gravity *G*_1_ of the sample is obtained by using a spring dynamometer, then the sample is completely immersed in water. The spring dynamometer shows *G*_2_, V is the volume of the overflow water and the volume of the sample, $\rho_{w}$ is the density of water, the buoyancy *F* of the sample in water is as follows:

(43) $$F=G_{1}-G_{2}=\rho_{w}Vg$$

From Equations (34) and (43):

(44) $$\rho =\frac{G_{1}\rho_{w}}{\left(G_{1}-G_{2}\right)}$$

This traditional method is used to measure the density of 11 kinds of rubber materials respectively.

Using the JAPAN-600C ultrasonic pulser/receiver, the contact probe has a 1 MHz frequency, and the densities of 11 kinds of rubber materials are determined based on the SRM. The buffer is PMMA. The reflectance of the buffer to the sample is obtained by the transmitted wave A_0_ and the surface reflected wave A, and the acoustic impedance *Z*_2_ of each material is obtained from Equation (41). Additionally, the sample density value can be obtained from Equation (42). Table 1 shows the comparison with the traditional density measurement results.

The density calculation error is 10.2% or less. Since the calculation error of the acoustic impedance based on the surface method is the same, it can be improved by correcting the error caused by the water layer in the previous chapter.

## 5. Conclusions

The measurement of the surface reflectivity of the sample and the buffer was standardized with reference to the reflected wave at the bottom of the buffer when there was no sample. The simulation method and experimental verification were used to calculate the storage modulus, loss modulus, and loss tangent by the BRM and SRM, and the results were then compared. The results show that the SRM is slightly larger than the traditional BRM, but it proves the feasibility of the surface reflection method for viscoelastic evaluation. When considering the influence of the thickness of the coupling dielectric layer on the result, the error between the two detection methods is reduced, and the optimization effect is obvious. Finally, the density measurement of the polymer material is based on the principle of the SRM, so the evaluation of the viscoelasticity of the material is more efficient and practical.

## Figures and Tables

**Table 1:** Density calculated by reflection coefficient (longitudinal wave 1 MHz). Sample materials are represented by sample name (column 1), among which EPX series belongs to the EPDM material mentioned above, OR series belongs to SBR1 material, and ORHS series belongs to SBR2 material. They are all named according to the hardness of the material, the physical properties of the filling material, etc.

Materials	Density by Reflection Coefficient (g/cm^3^)	Density by the Archimedes Method (g/cm^3^)	Difference (%)
EPX24	0.90	0.93	2.9
EPX46	0.95	0.98	3.2
EPX45	0.98	1.02	3.6
OR3C	0.98	1.04	5.5
OR3Si	1.03	1.07	3.7
OR4C	1.01	1.06	4.6
OR4Si	1.05	1.08	2.6
ORHS3C	0.93	1.03	9.7
ORHS3Si	0.93	1.02	8.8
ORHS4C	0.95	1.05	9.5
ORHS4Si	0.96	1.07	10.2
